# Effective use of biosensors for high-throughput library screening for metabolite production

**DOI:** 10.1093/jimb/kuab049

**Published:** 2021-08-04

**Authors:** Jennifer A Kaczmarek, Kristala L J Prather

**Affiliations:** Department of Chemical Engineering, Massachusetts Institute of Technology, Cambridge 02142, USA; Department of Chemical Engineering, Massachusetts Institute of Technology, Cambridge 02142, USA

**Keywords:** Biosensors, High-throughput screening, Metabolic engineering, Transcription factor, Directed evolution

## Abstract

The development of fast and affordable microbial production from recombinant pathways is a challenging endeavor, with targeted improvements difficult to predict due to the complex nature of living systems. To address the limitations in biosynthetic pathways, much work has been done to generate large libraries of various genetic parts (promoters, RBSs, enzymes, etc.) to discover library members that bring about significantly improved levels of metabolite production. To evaluate these large libraries, high throughput approaches are necessary, such as those that rely on biosensors. There are various modes of operation to apply biosensors to library screens that are available at different scales of throughput. The effectiveness of each biosensor-based method is dependent on the pathway or strain to which it is applied, and all approaches have strengths and weaknesses to be carefully considered for any high throughput library screen. In this review, we discuss the various approaches used in biosensor screening for improved metabolite production, focusing on transcription factor-based biosensors.

## Introduction

One of the foci of metabolic engineering is the improvement and optimization of metabolite production from microbial organisms (Stephanopoulos, 2012). There have been many advances toward discovering new target molecules that can be produced *de novo*; however, the productivities from these pathways is often suboptimal (Dietrich et al., 2013). Due to the complexity of production in living organisms, rational engineering of pathway improvement is commonly met with limited success, making optimization a process that is usually time- and labor-intensive (Cobb et al., 2013). To bring about significant improvement in microbial productivity levels without the need for full knowledge of the complexities of functionally expressing a heterologous metabolic pathway in a living host at optimal levels, it is advantageous to generate large (and usually randomized) libraries of whole cells or key pathway enzymes.

In the creation of these libraries, more often than not, only a very limited subset of variants will show significantly improved performance. Depending on the diversification method and target sequence size, genetic libraries can reach sizes of 10^9^ variants (Hanson-Manful & Patrick, 2013), making high-throughput screening (HTS) an indispensable tool in library sorting (Dietrich et al., 2010). Traditional chemical quantification methods, such as mass spectrometry or chromatography, though well-defined and accurate, are time consuming and will reasonably allow for only a fraction of the generated variants to be tested. Moreover, while some products can be screened directly as they are inherently fluorescent (Savitskaya et al., 2019) or of a distinctive color (Yang et al., 2018), this is not common, making product quantification a large bottleneck in the discovery of improved metabolic pathways or chassis.

**Fig. 1:**
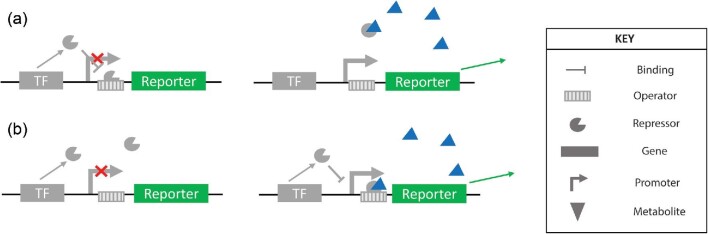
A schematic of transcriptional biosensors that are activated in the presence of the metabolite of interest. In (a), the transcription factor acts to repress the expression of the output gene until the repression is relieved by the metabolite. In (b), the transcription factor acts to activate the expression of the reporter gene only in the presence of the metabolite. The legend indicates the corresponding shapes for each respective part in the schematic.

Biosensors will detect internal stimuli such as pH (Zhu et al., 2019), cell density (Gupta et al., 2017), stress response (Polizzi & Kontoravdi, 2015), or metabolite concentration (Mannan et al., 2017), and produce a proportional response. They can be protein based, for example, relying on transcription factors (TFs) (Zhang et al., 2017) or fluorescent proteins (Ameen et al., 2016); or they can be nucleic acid based, utilizing riboswitches (Yang et al., 2013), including Spinach-based riboswitches (You et al., 2015). These tools allow for dynamic pathway control (Doong et al., 2018), environmental monitoring (Fernandez-López et al., 2015), or detection of changing levels of metabolite without the need for direct evaluation of titer (Rogers & Church, 2016). The development of biosensors has been crucial in the testing and screening of microbial libraries, greatly increasing the size of searchable library space as they enable the detection of inconspicuous small molecules. Biosensors allow for the bypassing of the lengthy and laborious product quantification step, increasing the speed and throughput of library screening. The most commonly utilized form of biosensors for these applications is the TF-based biosensor (Fig. 1), where the output is controlled via transcriptional regulation, and regulation is coordinated by a TF that is responsive to the presence of the target molecule (Schallmey et al., 2014).

When generating libraries, there are often standard diversification methods to achieve a desired outcome, such as error-prone PCR (McCullum et al., 2010) for randomized enzyme libraries, transposon insertion (Cain et al., 2020) for whole-cell genetic disruption libraries, or atmospheric and room-temperature plasma (ARTP) for whole-cell libraries from DNA damage (Zhang et al., 2014). These methods tend to be pathway independent and are able to achieve the desired diversity for a host of applications with only minor methodological modification. Conversely, biosensor screens are much more specific and tend to require fine tuning for effective application to the given pathway. It is possible to adjust the conditions of the screen (Flachbart et al., 2019), the method of screening used (Kortmann et al., 2019), or the TF itself, where TFs are often mutated to allow for novel product detection (Taylor et al., 2016) or for improved dynamics (Mannan et al., 2017). To date, much effort has gone into the development of biosensors to facilitate successful high throughput library screening. There is thus a wealth of work reported on tuning, modifying, and evolving biosensors that has been recently reviewed elsewhere (Koch et al., 2019; Lim et al., 2018; Lin et al., 2017) and will not be detailed in this review.

**Fig. 2:**
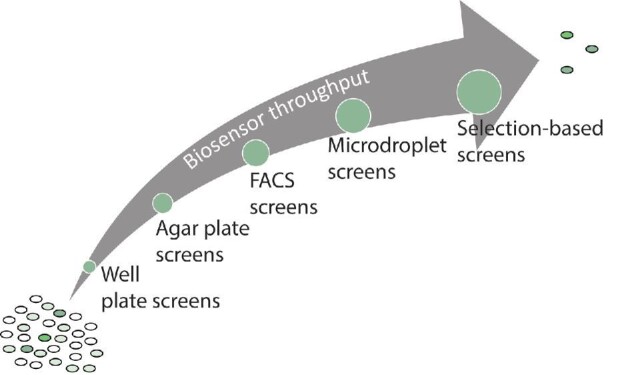
The screening assays used in biosensor-mediated high-throughput screening (HTS) allow for the selection of improved variants in a library based on an easily detectable output, such as fluorescence. The throughput for each assay differs and impacts the size of searchable library space in HTS.

Biosensors offer great potential, but achieving this potential requires correct selection of screening method and a biosensor that has (or can be modified to have) the operational range to detect the desired product. The main biosensor screen modalities are well plates, agar plates, fluorescence-activated cell sorting (FACS), droplet-based screening, and selection-based methods; each approach has a different capacity for library size (Fig. 2). While throughput is one of the most important considerations when applying a biosensor to a library screen, there are other aspects that are application and biosensor specific. Sensor characteristics, equipment requirements, risk of false positives that pass through the screen, and the dynamics of product generation and transport are all key factors beyond the throughput of the assay that should be weighed when selecting the method for a successful biosensor-assisted HTS campaign.

In this review, we will discuss the various ways in which biosensors—specifically TF-based biosensors—have been applied in high-throughput library screens (Table 1), as well as any modifications made to overcome challenges to the success of these screens. We will highlight the advantages and disadvantages of each method as well as the constraints that may prefer one screen over another.

**Table 1.** Biosensors Applied to Improve Product Yields

Screen method	Organism	Target molecule	Library type	Highlighted improvements	Ref.
Well plate	*E. coli*	Vanillin and syringaldehyde	Metagenomic library	Discovery of 147 new clones that selectively degrade lignin	(Ho et al., 2018)
	*E. coli*	Isobutanol	ARTP whole-cell library	2-fold improved production relative to base strain	(Yu et al., 2019)
	*E. coli*	Glucaric acid	Degenerate nucleotide-generated enzyme library	4-fold improvement in specific titer relative to parent strain and 2.5-fold increase in *k_cat_/K_m_*	(Zheng et al., 2018)
	*Y. lipolytica/E. coli*	Erythritol	ARTP whole-cell library	2.4-fold improved production relative to original strain	(Qiu et al., 2020)
Blue-white agar plate screen	*E. coli*	Salicylate	RBS library/transposon-mediated mutagenesis	123% increased production in shake flask	(Qian et al., 2019)
	*E. coli*	Mevalonate	RBS library	3.8-fold improved production relative to original plasmid	(Tang & Cirino, 2011)
	*E. coli*	Triacetic acid lactone	epPCR and SSM libraries	19-fold improved catalytic efficiency of 2-pyrone synthase	(Tang et al., 2013)
	*E. coli*	Resveratrol	epPCR enzyme library	1.8-fold improved specific enzyme activity over WT	(Xiong et al., 2017)
GFP-based agar plate screen	*E. coli*	Lactulose	epPCR enzyme library	∼32-fold enhanced expression of C2E enzyme	(Wu et al., 2017)
FACS	*E. coli*	Acrylic acid	epPCR enzyme library	1.6-fold improved RAPc8 amidase *k_cat_/K_m_*	(Raghavan et al., 2019)
	*S. cerevisiae*	*cis, cis*-muconic *acid*	UV-mutagenesis whole-cell library	49.7% increased production compared to control strain	(Wang et al., 2020)
	*E. coli*	3-dehydroshikimate (DHS)	ARTP mutant library	90% increased production over base strain	(Li et al., 2019)
	*S. cerevisiae*	Fatty acyl-CoAs	Whole-cell gene overexpression library	80% increased fatty alcohol levels over base strain	(Dabirian et al., 2019)
	*C. glutamicum*	L-lysine	epPCR enzyme library	Up to 19% increased titer from plasmid; up to 14% increased titer from chromosomal pathway expression	(Kortmann et al., 2019)
	*C. glutamicum*	L-valine	ARTP whole-cell library	21.5% increased production compared to starting strain	(Han et al., 2020)
	*C. glutamicum*	Shikimic acid	RBS library	90% increased production compared to production using a known strong RBS	(Liu et al., 2018)
	*C. glutamicum*	L-arginine, L-Histidine, L-lysine	epPCR enzyme library	87-fold improved L-arginine production, 37-fold improved L-lysine production and 17 mM L-Histidine (no production from WT)	(Schendzielorz et al., 2014)
	*C. glutamicum*	L-methionine, L-valine, L-leucine, L-isoleucine	MNNG whole-cell library	Up to 8 mM L-valine, 2 mM L-isoleucine, 1 mM L-leucine from *C. glutamicum* (no production from WT strain)	(Mustafi et al., 2012)
	*M. extorquens*	Mevalonate	epPCR library	2.8-fold improved yield	(Liang et al., 2017)
	*S. cerevisiae*	Malonyl-CoA	cDNA library	120% increased production relative to WT	(Li et al., 2015)
	*C. glutamicum*	L-serine	ARTP whole-cell library	35.9% increased titer compared to the parent strain	(Xin Zhang et al., 2018)
	*E. coli*	Ectoine	epPCR enzyme library	4.1-fold improved EctB *k_cat_*/*K_m_*	(Chen et al., 2015)
	*C. glutamicum*	L-Histidine	MNNG-generated whole-cell library	Up to 0.7 mM production (over 0 mM from base strain)	(Della Corte et al., 2020)
	*E. coli*	L-phenylalanine	MNNG-generated whole-cell library	4.3-fold improved production compared to WT	(Mahr et al., 2016)
	*E. coli*	*trans*-cinnamic acid (CA)	epPCR enzyme library	10 to 60% more CA or *p-*coumaric acid from L-phenylalanine or L-tyrosine, respectively	(Flachbart et al., 2019)
FADS	*E. coli*	3-dehydroshikimic acid	ARTP whole-cell library	21% improved DHS relative to previous mutant (Liu et al., 2018)	(Tu et al., 2020)
Selection-based (agar plate)	*C. crenatum*	L-arginine	ARTP whole-cell library	13.5% increased production relative to an engineered *C. crenatum* strain (Man et al., 2016)	(Xu et al., 2020)
	*E. coli*	L-phenylalanine	ARTP whole-cell library	160.2% improved L-phenylalanine from parental stain	(Liu et al., 2017)
Selection-based (liquid culture)	*E. coli*	1-butanol	RBS library	35% increased production relative to original pathway	(Dietrich et al., 2013)
	*E. coli*	3-hydroxypropionic acid	Assembly PCR-generated enzyme library	25% increased production relative to original pathway	(Seok et al., 2018)
	*S. cerevisiae*	*cis, cis*-muconic *acid* (CCM)	Multicopy gene insertion library	Over 2 g/l in bioreactor from a parental strain that previously showed no quantifiable produciton	(Snoek et al., 2018)
	*E. coli*	L-tryptophan	epPCR enzyme library, ARTP whole-cell library, site saturation mutagenesis library	65% increased production relative to WT	(Liu et al., 2017)
	*E.coli*	Glucaric acid	MAGE library	22-fold improved production relative to parent strain	(Raman et al., 2014)
	*E. coli*	Naringenin	MAGE library	36-fold improved production relative to parent strain	(Raman et al., 2014)
Auxotrophic screens—microtiter plate	*E.coli*	Isobutanol	NTG whole-cell library	5-fold improved production relative to parent strain	(Saleski et al., 2019)
Auxotrophic screens—droplet based	*E. coli*	Isobutanol	Transposon-mediated insertion library	Chromosomal expression on order of plasmid-based expression in top performing library member	(Saleski et al., 2021)

ARTP: atmospheric and room-temperature plasma; MNNG: N’-methyl-N’-nitro-N-nitrosoguanidine; MAGE: multiplex automated genomic engineering; NTG: N-methyl-N’-nitro-N-nitrosoguanidine.

### Well Plate-Based Biosensor Applications

Well plate assays are carried out in micro- or deep-well volumes (200 to 1000 μL), where each library member is compartmentalized and an average output, frequently fluorescence, is quantified for each volume. This method is on the lower end of high throughput and requires significant experimental effort in the setting up and inoculation of plates with each library member. The use of individual wells for each library member has the benefit of separating each within its own well, ensuring that variation at the single-cell level does not influence the outcome of the screen. However, with a lower throughput (∼10^4^), there is a limit to the searchable library space. That is not to say that this method cannot be successful, as it has been used to improve production of isobutanol (Yu et al., 2019) via strain evolution and used to discover 147 strain variants that could selectively degrade lignin to vanillin and syringaldehyde from a metagenomic library (Ho et al., 2018).

Zheng et al. applied a plate-based assay that separated the biosensor strain from the production strain in a “two-strain one-pot” method (Zheng et al., 2018). This work found that there was more reproducibility when the production and biosensor vectors were located within different strains compared to a single strain containing multiple vectors. The producing cells were independently grown and their supernatants were used to supplement the medium for a biosensor grown in parallel, with the relative amounts of product present in the supernatant activating each biosensor culture accordingly. Using this procedure, the authors successfully screened a library of *myo*-inositol oxygenase variants in *Escherichia coli* to discover three different mutants that exhibited improved glucaric acid production. The identified variants displayed *k_cat_/K*_m_ values over twice that of the wild-type and showed improved *V_max_* values. The screening system was also applied to yeast (though used for pathway optimization rather than library screening) to screen for higher producers, indicating the ability of this method to pair eukaryotic cell production with a separate prokaryotic biosensor screen. This method was carried out recently by Qiu et al. (2020) when they used a similar method to pair a bacterial EryD-based sensor with an ARTP-derived genomic library in *Yarrowia lipolytica* to screen for improved erythritol production from yeast.

The use of a well plate-based screen is useful if a lower throughput can discover improved variants or if the lower throughput can be maximized with the use of robotic systems designed for handling large numbers of samples. Further, it may be necessary to use this type of screen if the product is known to freely diffuse into nonproducing cells or if it is preferred that the biosensor and the production strain be cultured separately from one another.

### Agar Plate-Based Biosensor Screens

In agar plate-based screens, the biosensor output is typically fluorescent or colorimetric, and the library containing the biosensor is plated and visually inspected to identify high producers. Agar plate screens are less laborious and slightly higher throughput compared to well plate screens. A challenge in implementing this method is the careful tuning of the sensitivity and output range of the biosensor that is required to facilitate the detection of higher producers. With inspection being done by eye, high-producing colonies must show a clearly distinguishable output in response to product. This obstacle comes with the benefit of a relatively straightforward screening procedure that does not require specialized equipment. Agar plate-based screening by Wu et al. used a modified form of LacI that was sensitive to lactulose, an analog of lactose (Wu et al., 2017). This work relied on a biosensor with GFP as the signal output to discover high-producing enzymes from a library of cellobiose 2-epimerase mutants. The top performing mutant was found to exhibit 22-fold higher lactulose titer and 32-fold increased expression.

Though many biosensors used in screening campaigns are based on fluorescence, an alternative is the use of blue-white screen with *lacZ* replacing *gfp* as a reporter gene. In this configuration, LacZ production is controlled by a metabolite-inducible promoter and colonies turn blue in the presence of 5-bromo-4-chloro-3-indolyl-β-D-galactopyranoside (X-gal) as long as there are sufficient levels of metabolite. This screen has been used in combination with biosensors based on mutated forms of the TF AraC. AraC naturally responds to L-arabinose, but was evolved to create the biosensors AraC-mev (Tang & Cirino, 2011), AraC-TAL (Tang et al., 2013), and AraC-SA (Qian et al., 2019) to screen enzymatic and whole-cell libraries producing the small molecules mevalonate, triacetic acid lactone (TAL), and salicylate, respectively. The blue-white screen was also employed to screen an error-prone PCR library of *p*-coumarate: CoA ligase (4CL) variants for improved resveratrol production (Xiong et al., 2017).

While agar plate-based library screening can be less laborious than a well plate-based assay, this method is unlikely to be suitable for all biosensors and libraries as significant differences in biosensor signal are generally necessary for visual detection. Determining the operable range to achieve this may not be possible or could require extensive evolution and/or modification of the biosensor itself. Nevertheless, this method can be useful when screening on solid media is more beneficial or when specialized equipment (i.e., microplate detectors and cell sorters) is not accessible.

### FACS-Based Biosensor Screens

Currently, FACS is the most frequently utilized method for biosensor-mediated HTS. Fluorescence-activated cell sorting analyzes large libraries of variants at the single-cell level, separating cells that exhibit the highest fluorescence from a bulk culture. Sorted cells are then subjected to a secondary, lower throughput screen to verify that the subset that passed the screen are true improved variants. Using FACS, it is possible to screen libraries that reach sizes of ∼10^6^ in the search for improved variants. As this method is commonly used and the equipment for FACS is well defined, there is a fairly standard screening procedure including: diversity generation, FACS screening, and “hit” verification through secondary screening (Binder et al., 2012). Challenges in applying this screen can arise due to variation at the single-cell level and the diffusion of products from one cell to the next, both of which can lead to false positives within the screen. Even with these obstacles, there are a number of reports of successful use of such biosensors in combination with FACS to discover enzyme variants and whole-cell mutants that yield improved production in both bacterial (Chen et al., 2015; Della Corte et al., 2020; Han et al., 2020; Kortmann et al., 2019; Li et al., 2019; Liu et al., 2018; Mustafi et al., 2012; Raghavan et al., 2019; Schendzielorz et al., 2014) and yeast (Dabirian et al., 2019; Li et al., 2015; Wang et al., 2020) hosts.

Biosynthetic pathway enzymes are obvious targets for mutation and screening to improve the production of target metabolite; however, mutations in the broader host genome—affecting both metabolism and resource allocation—can also prove beneficial. One such example involved the generation of a QscR library for improved mevalonate production from methanol in *Methylobacterium extorquens* AM1 (Liang et al., 2017). QscR is a regulatory element known to act upon the serine cycle to control carbon flux to enable growth on methanol. By mutating this enzyme and screening for high producers in a single round of directed evolution, a regulator variant that led to a 60% increase in mevalonate titer was identified. This study highlighted that there is much to gain from screening targets that indirectly influence the pathway.

In FACS, false positives often arise due to cells that are larger and more fluorescent, or due to heterogeneity in the expression of GFP or the pathway enzymes at the single-cell level. Following FACS screens, false positives are filtered out by a form of enrichment. Whether this comes in the form of further rounds of FACS screening, agar plate screening, or screening in a well plate, this secondary screen is often necessary to find the true improved variants. Florescence-activated cell sorting relies on gating, that is, setting boundaries to separate cell populations based on the plots generated from flow cytometry data for appropriate sorting, and these boundaries have an impact on the screen. To address whether the gating strategy employed impacts the rate of false positives or the titer produced from the final improved variants, Kortmann et al. compared two different techniques in FACS (Kortmann et al., 2019). The first gate was chosen to maximize the exclusion of the base strain based on a diagonal between forward scatter (a proxy for size) and fluorescence. By following this diagonal, size was considered to avoid sorting cells that were large and thus more fluorescent. The other gating technique did not consider the size, but only sorted cells based on their fluorescence, choosing the top percentage based on a histogram of fluorescence. While the second gate would have a bias toward larger cells, both gates led to the discovery of improved mutants, with similar numbers of false positives resulting from both sorts. Overall, one mutant from each sort was chosen to test via plasmid-based expression and chromosomal integration, both showing similarly improved titers, indicating that the chosen gating method had no significant impact on the outcome of the screen.

The secondary screen will only ensure that the selected variants are true positives, and will not address the decrease in biosensor effectiveness due to false positives. When biosensors are characterized within a clonal population, they very often show a clear and dose-dependent average fluorescent response to the target as there is inherent averaging across the population. However, such well-performing sensors based on their average fluorescence value in a population may not always translate to a biosensor that is effective at the single-cell level where heterogeneity in cell size and heterologous protein production have a larger effect. Diffusion of product between cells also allows for the emergence of “cheaters” as product freely enters lower-producing cells from higher-producing cells in culture prior to cell sorting (Dietrich et al., 2010). These challenges may be part of the reason that there is a great wealth of literature detailing the development and characterization of biosensors, but relatively less when it comes to effectively using them in library screening—particularly at the single-cell level (Flachbart et al., 2019).

To address these issues, there has been work aimed at preventing false positives rather than just removing them from consideration after they occur. To address product diffusion, Woolston et al. (2018) added glutathione—a formaldehyde scavenger—as a “sink” for formaldehyde, their sensed molecule. This enabled the sensor to clearly separate different populations of cells as glutathione formed a complex with formaldehyde before it could diffuse from producing to nonproducing cells.

In working to decrease culture heterogeneity, Flachbart et al. added an *araE* gene to their system to ensure homogenous *xal_TC_* expression from an L-Ara-inducible promoter. This modification ensured all or nothing gene expression, as was previously demonstrated in decreasing single-cell variation in Xal_TC_ output (Khlebnikov et al., 2002). To reduce diffusion within the library prior to sorting, the inoculum density and culture time were optimized. Using these techniques, a biosensor was successfully applied to the discovery of an improved Xal*_TC_* enzyme for *trans*-cinnamic acid production (Flachbart et al., 2019).

Overall, the reports of success using FACS indicate that it is a promising and straightforward screening method to choose for most applications. The benefits of the high throughput and relative simplicity of this screen come at the risk of a less efficient screen due to heterogeneity at the single-cell level or diffusion of the product into low-producing cells. Even so, this method is still quite useful and can generally be applied in most desired screens where there is a clear difference in output signal for different levels of product titer *and* when the product diffusion is not of concern based on the timescale or the screen or can be counteracted by other methods.

### Droplet-Based Screening and Selection

Droplet-based screening methods encapsulate members of the library of interest within a set of droplets for HTS (Jang et al., 2016). Depending on the composition of the droplets, screening can take place via either fluorescence activated droplet sorting (FADS) (Tu et al., 2020) when the external layer of the droplet is hydrophobic (i.e., water/oil emulsions) or through microdroplet FACS (Siedler et al., 2017) when the external layer is not hydrophobic (e.g., water/oil/water emulsions). Fluorescence activated droplet sorting often utilizes specialized microfluidic equipment for droplet sorting while FACS relies on traditional cell sorting equipment. Agarose beads have also been used as droplets to enable sorting via FACS (Ma et al., 2019), but this particular application has yet to be applied to *in vivo* biosensor screens. Similarly, many other applications of droplet-based sorting do not rely on *in vivo* biosensors and instead involve encapsulation of cellular lysates containing the enzyme to be improved and a fluorogenic substrate (Kintses et al., 2012).

Similar to the well plate-based method, a benefit of this method is the ability to combine a bacterial biosensor and a yeast production strain within a single droplet. Transcription factor-based biosensors often rely on prokaryotic TFs as they tend to be much simpler than eukaryotic systems (Zhang & Shi, 2021). While there has been some success in translating prokaryotic systems into yeast and some systems that utilize native yeast regulation, there is limited screening capacity for yeast libraries (Li et al., 2015). By using separate sensing strains that are more conducive to the transcriptional regulation machinery, a greater range of products and production chassis can be targeted for improvements.

Droplet-based sorting has been shown to be more effective than FACS-based sorting for finding improved library members. Tu et al. demonstrated that, when compared to FACS, their biosensor-based FADS had an improved enrichment rate over the traditional FACS sort (Tu et al., 2020). Though using a fluorescent product and not a biosensor, Wagner et al. showed that droplet-based sorting was much more useful for products that are secreted, as FACS will preferentially sort high intracellular producers (Wagner et al., 2018).

While the droplet-based sorting method combines the advantages of FACS and microplate assays with both high throughput and compartmentalization, it also suffers from the cell-to-cell variability present in FACS, where heterogeneity within the cultures can impact the overall screen. Beyond this disparity on the cellular level, there is also variability in the encapsulation process itself. In droplet-based screens, cells are encapsulated based on a Poisson distribution and the number of cells in each droplet cannot be directly controlled. To avoid false positives due to multiple production cells within one droplet, encapsulation densities are often rather low, such that it is possible for only one in 10 droplets to have a single cell (still with one in 100 droplets having two cells) (Abatemarco et al., 2017). Overall, there have yet to be significant applications of droplet-based sorting using *in vivo* biosensor screens, but as the technology and the ability to control encapsulation develops, this method may be more widely adopted. If the necessary microfluidic equipment is available, this screening method could be beneficial when product diffusion cannot be addressed by other means but a higher throughput is still desired.

### Selection-Based Biosensor Screening

One of the major advantages to using a selection-based biosensor screen is the theoretical throughput. Using this screening modality with proper biosensor tuning, library size is only limited by the efficiency of the transformation method, as nonproductive strains should simply not grow, enabling growth of all higher producers (Dietrich et al., 2010). In this method, the biosensor output correlates target product levels to cellular fitness, often through the expression of a selectable marker, linking the production of inconspicuous metabolites to growth levels. A selection-based screen can be carried out in a liquid culture or on solid media. However, as with every other method, it exhibits drawbacks that limit its potential. First, there is the risk of enriching evolutionary escapees (Raman et al., 2014) as a result of adaptive mutation in the host cell. These cells will gain adaptations that allow them to survive the selective pressure without the desired productivity improvements. Another challenge, though one that can be addressed with proper biosensor tuning, is that even a slightly leaky biosensor output level could lead to survival of low producers or even nonproducers out of the library, increasing false positive risk and decreasing the performance of the screen (Dietrich et al., 2010).

Selection-based biosensors exhibit significant improvement relative to selecting variants from an unscreened control group, as Liu et al. showed when applying their streptomycin-resistance-based sensor to a randomly generated strain library. They found that 69% of mutants that were selected produced significantly higher titers of L-phenylalanine relative to only 2% that produced higher titers in the unscreened control group. The final mutant showed 160% increased production relative to the parental strain (Liu et al., 2017). Further examples of successful applications of this method include the discovery of mutants for improved production of muconic acid (Snoek et al., 2018), 1-butanol (Dietrich et al., 2013), and 3-hydroxypropionic acid (Seok et al., 2018).

While the sensor-selectors mentioned above are based on antibiotic resistance, this is not the only method of selection, and there are notable examples of using other methods. Rogers et al. developed multiple sensors for pathway improvement based on regulation of an outer membrane protein, TolC, that removes toxic SDS (Raman et al., 2014). A further benefit to this system was the ability to run iterations in which positive and negative selection were alternated to remove evolutionary escapees. In this work, evolutionary escapees were the cells that survived the screen through acquisition of a mutation that enabled constitutive expression of *tolC*. To remove these escapees, a toggled selection approach was used: the expression of *tolC* confers sensitivity to Colicin E1, meaning that the application of this bacteriocin selected against any runaway mutants present following the SDS screen. A biosensor screen based on sensitivity to sucrose allowed for the detection of mutants with increased L-arginine production in *C. crenatum* (Xu et al., 2020). In this work, L-arginine levels above 60 mM would repress the output of SacB—whose expression is lethal to cells in 10% sucrose—in *Corynebacterium crenatum*, linking higher levels of survival to lower outputs of SacB caused by increased levels of L-arginine.

A different technique relied upon to the ability to utilize maltose as the driver for selection. In this method, maltose was provided as the sole carbon source, with a crucial step in the maltose utilization pathway removed from the host. The gene encoding the missing enzyme, *malQ*, was then expressed from an L-tryptophan-responsive promoter which generated strains that were directly dependent upon L-Trp concentration for growth on maltose, directly correlating survival to L-Trp levels. This method was shown to exhibit a significantly reduced chance of evolutionary escape when compared to a traditional antibiotic-resistance-based screen. TrpE mutants leading to improved titers were discovered from both enzyme and whole-cell libraries increased L-Trp production (Liu et al., 2017).

The principles used in selection- and droplet-based screens can be combined when screening for high levels of survival based on complementary feeding within a microdroplet. This approach was successfully applied through syntrophic co-culture amplification of production (SnoCAP), which was used to sort both whole-cell libraries made through mutagenesis (Saleski et al., 2019) and transposon-mediated insertion libraries (Saleski et al., 2021) for improved isobutanol titers. The first application of SnoCAP used NTG to mutagenize the host strain and demonstrated the application of their novel screening method to find an optimal strain for isobutanol production. In the follow-up publication, transposons were used to transfer parts of the biosynthetic pathway in random sites of the chromosome, probing the effects of genomic location of homologous pathway enzymes on production. Syntrophic co-culture amplification of production screening discovered a strain that expressed all homologous enzymes in the host genome that exhibited yields similar to those from a plasmid-based system.

Syntrophic co-culture amplification of production relies on a coculture method to discover improved variants through a metabolic cross-feeding circuit enabling commensal growth of a sensor and secretion strain. The sensor strain constitutively produces GFP and is also auxotrophic for the product from the secretion strain. When there is less product, the GFP-expressing strain grows less, leading to lower fluorescence levels. This means the top producers will fluoresce at higher levels, enabling HTS for improved library members. In the original SnoCAP publication, the method was demonstrated using microtiter plates, agar plates, and droplets with the microtiter and agar plates being used for mutational library screening. In the application of SnoCAP to random pathway insertion libraries, both droplet and microtiter plate assays were used in screening.

With most library generation methods commonly resulting in only small fractions of high producers, the use of a selection-based screen allows for extremely large libraries to be analyzed as, typically, only these high producers will survive. This method is useful for applications where compartmentalization is desired (for the agar-plate format), extremely high throughput is needed, or equipment limitations exist. However, this comes at the hazard of evolutionary escape, a need for sufficient production rates such that any cells are able to survive the selective pressure, and a well-tuned biosensor.

## Conclusion

Biosensors have enormous potential across metabolic engineering applications with widespread application to directed evolution—a technique that could be used to discover new catalysts and to improve upon existing ones. However, once a biosensor has been assembled, characterized, and tuned, this does not guarantee its successful application to a screen. There is much work and consideration required when applying a biosensor to the desired screen as different screening methods will be more or less well suited for a given application. It is possible that the constraints of one screen may diminish the capacity of the biosensor to screen for the metabolite of interest and that another screen can be applied successfully or modifications can be made to the desired screen to circumvent these hindrances. As technology and methodology continue to develop, there is significant promise that these pitfalls will be addressed, allowing for screening systems with both high throughput and low false positives. With more effective HTS technology, the ability to optimize new pathways that continue to be discovered will allow maximum titers to be attained with minimal cost.
